# ‘As His was Not a Surgical Case it was Not My *Duty* to Attend Him’: The Surgeon’s Role in the Nineteenth-Century Royal Dockyards

**DOI:** 10.1017/mdh.2013.39

**Published:** 2013-10

**Authors:** Richard Biddle

**Affiliations:** University of Oxford, 45–47 Banbury Road, Oxford OX2 6PE, UK

**Keywords:** Royal dockyards, Surgeon, Royal Navy, Healthcare, Dockworkers, Shipbuilding

## Abstract

Despite a varied historical literature on the nineteenth-century royal dockyards, very little has been written about the health issues associated with naval shipbuilding or the healthcare facilities that were provided for dockworkers in the period. This article focuses mainly on the latter. Drawing on archival sources from the home dockyards, an examination is made of the duties and responsibilities of dockyard surgeons. These are found to have expanded considerably as healthcare provision became steadily more comprehensive. It is argued that as providers to a civilian workforce, the naval authorities were in the vanguard when it came to implementing perceived advances in medical practice. It is also contended, however, that while many dockworkers benefited as a result, this positive appraisal needs to be set against the more ambiguous aspects of the surgeon’s role. Although surgeons treated the sick and injured, their growing prominence in other dockyard matters, such as retirement and the policing of sickness, is shown to have created tension in their relationship with the workforce.

The nineteenth-century royal dockyards were enormous state-run establishments, headed by a senior naval officer but with a civilian workforce. In the major naval dockyard towns of Chatham and Plymouth they were employing around 3500 men and boys by the 1860s; in Portsmouth the figure was closer to 5000.^1^ At any given time this could amount to as much as seventy per cent of the male industrial population.^2^ The shaping of local economies was not their only influence.^3^ Physically these vast industrial complexes dominated the landscape, and continued to do so throughout the century, as changes to ship design and construction led to many of the yards being extended. By the time that the ‘Great Extension’ at Portsmouth was finished in 1881, the dockyard occupied a 261-acre site, with three colossal basins having been added to its existing array of docks, factories and foundries.^4^ The yards were also dangerous places. The presence of thousands of people working in close proximity to deep water, precipitous dry docks and highly combustible materials, along with the movement of heavy loads across the site and a myriad of other similar factors, all made them hazardous places to work in. Charles Dickens very aptly captured the royal dockyards’ capacity to inspire the Victorian imagination. While conversing with a boy on the shores of the Medway, his Uncommerical Traveller remarked: ‘[H]e several times directed his eyes to one distant quarter of the landscape, and spoke with vague mysterious awe of the “Yard”’.^5^

For a global maritime power like Britain in the nineteenth century, the royal dockyards were vital to the national interest.^6^ Thus, unsurprisingly, they have attracted considerable scholarly attention. Much of this has focused on the technical aspects of the yards and the ships that they built and maintained.^7^ Their workforce has also figured in discussions about labour and labour relations.^8^ This article, however, is concerned with healthcare provision in the royal dockyards and in particular with dockyard surgeons. Drawing on evidence from the home yards, mainly in the form of surgeons’ letter books and casebooks, it outlines the duties and responsibilities of these medical officers, and traces how they changed during the course of the century. As well as shedding light on surgeons’ complicated relationships with dockworkers, the richness of the sources, which cover everything from mundane requests for stationery to in-depth medical reports and orders from the medical department of the Navy, also offer broader insights into the naval authorities as healthcare providers to a civilian workforce.

The areas of health and medicine in the nineteenth-century royal dockyards remain very under-researched. This is a rather curious state of affairs, given that the Royal Navy’s ability to protect overseas trade and the Empire ultimately depended upon the proper functioning of these establishments. Lloyd and Coulter in their volume of the seminal four-volume work *Medicine and the Navy* have little to say on either matter, other than to note that from 1881 naval surgeons from the Medical School at Haslar Hospital visited Portsmouth Dockyard to learn about naval hygiene in relation to ship construction.^9^ Research is similarly lacking in respect of commercial docks and shipbuilders in the period.^10^ In his exhaustive study of the Seaman’s Hospital Society, Gordon Cook mentions that admissions to the hospital for merchant sailors at Albert Dock in London included people involved in accidents at the docks, although he does not elaborate further.^11^ More recently Margaret Makepeace has drawn attention to the fact that London dock companies made allowances for employees injured at work. But, as her aim was to understand and evaluate the strategies used by the East India Company in the management of its warehouse labourers, she provides no more detail.^12^

Until a few years ago, D.S. Wright was the only scholar to have published specifically in the subject area. Back in 1968, his study of the medical facilities at Chatham Dockyard appeared in the *Journal of the Royal Navy Medical Service*.^13^ In surveying their development over a 350-year period, Wright emphasised notions of progress and the contribution made by medical personnel. As a serving medical officer at Chatham at the time, this was entirely understandable, and reflected his own interests and those of his likely audience. His subsequent work was written very much with the healthcare professional in mind. Although always referring to the dockyards in the past, Wright invoked their history as a way to contextualise and provoke discussion about modern issues in occupational health.^14^ More recently, the author of the present article has contributed to the field. However, publications to date have been more from the perspective of dockworkers as opposed to surgeons and have specifically considered how the health of the former was affected by the transition in naval shipbuilding from sail and wood to steam and iron.^15^ So, in many respects what follows treads fresh ground, albeit that some of the themes which emerge will be seen to have relevance to broader debates in the history of medicine.

## The Qualifications and Experience of Dockyard Surgeons

Thankfully, the naval authorities were not as slow as historians to recognise the importance of dockworkers’ health. At Chatham Dockyard, reference to the provision of a surgeon can be traced back to the beginning of the seventeenth century,^16^ while at Portsmouth it is known that from 1665 the surgeon had a house located on-site as part of his remuneration.^17^ By the nineteenth century, healthcare provision had expanded somewhat. In the larger yards, the Medical Departments (as they were referred to after 1826) usually comprised a surgeon, an assistant or assisting surgeon, along with a messenger.^18^

The post of dockyard surgeon was usually filled directly from the Navy.^19^ Dockyard appointments were highly sought after and, as the *Navy Lists* show, they invariably went to experienced men.^20^ The two examples that follow are indicative and illustrate this well. Dr William Gunn MD was appointed to Sheerness Dockyard in June 1855, after having been a naval surgeon for twenty years, during which time he served on a variety of vessels in both the home and foreign stations. He had also been a surgeon and medical storekeeper at Halifax Hospital (Canada) and at Deptford Victualling Yard. Gunn then moved to Chatham Dockyard in 1859, retiring six years later with the honorary rank of Deputy Inspector General of Hospitals and Fleets.^21^ Dr Edward Cree MD was similarly experienced. By the time of his appointment to Portsmouth Dockyard in 1864 he had twenty-seven years’ service. This included ten years in the Far East where he saw action in the First Opium War. He also served in the Crimean War, and was present at the capture of Sevastopol.^22^ As has been documented elsewhere, shipboard life presented medical officers with unique opportunities to enhance their knowledge and develop their skills.^23^ Even in times of peace, they were frequently called upon to fulfil the roles of surgeon, physician, dispenser and sanitary officer, usually without the benefit of discussion or advice from fellow practitioners.^24^ Hence, by the time a surgeon reached the dockyard he had a strong practical and theoretical grounding in the main branches of medicine, along with an understanding of preventative medicine and the importance of hygiene.

The calibre of dockyard surgeons was also reflected in their remuneration. In 1847, the post attracted an annual salary of up to £500.^25^ By 1868, a further allowance of £106 had been added to cover the costs of servants, fuel and light.^26^ Little wonder that they were attractive posts. Even without factoring in accommodation entitlements, this compared favourably with the income that might have been derived from private practice at this time. It should also be remembered that the dockyard surgeon’s salary was not dependent upon building and maintaining a caseload of paying clients; nor was it subject to the costs that this often involved.^27^ It is notable, too, that following the parliamentary commission of 1840 (which led to the rank of physician being abolished and the introduction of new grades of surgeon to the naval medical service) fresh appointments, particularly at the larger dockyards, were at the rank of staff surgeon.^28^ From the 1880s onwards such posts further increased in rank to that of fleet surgeon. By 1882, the medical department at Portsmouth Dockyard was headed by a fleet surgeon, who had either a staff surgeon or two normal surgeons under him.^29^ Evidently the naval authorities were not prepared to leave the health of their civilian workforce to chance.

## Patient Client Base and Caseload

Dockyard medical departments had their own on-site premises, which contemporaries referred to as the ‘surgery’. Detail about the dimensions of these buildings and their interior layouts is sparse. The best evidence is from Chatham where, by all accounts, the surgery was a small building, comprised of a surgeon’s room, a receiving room and a kitchen, where the range was kept going constantly in order to supply hot water.^30^ It is clear from Chatham and the other yards that these surgeries were not equipped to deal with inpatients. Where this type of care was necessary, or when an injury prevented a patient from walking home, surgeons usually arranged for a cab at the dockyard’s expense. Alternatively, from April 1815, they had the option to send more serious cases for treatment at a naval hospital.^31^ So, for example, surgeons at Portsmouth Dockyard used Haslar Hospital, while those at Chatham and Sheerness used Melville Hospital. Use was also made of hospital ships. At Pembroke Dockyard patients requiring hospital treatment were sent to HMS *Nankin*.

A dockyard surgeon’s client base comprised a number of different groups, of which dockworkers were by far the largest. Throughout the nineteenth century all dockworkers were entitled to free healthcare for any injury or illness that was directly attributable to the dockyard.^32^ For the most part, this equated to physical injuries, although it should not be taken from this that all surgeons did was to patch up injured men. Very often complications such as infections set in. Many patients therefore required ongoing treatment, which often saw the surgeon taking on the role of physician in the management of such cases. In keeping with naval terminology, physical injuries were referred to as ‘hurts’.^33^ Hurts were divided into two categories: ‘slight’ and ‘serious’. Men designated as having a serious hurt were entered on the ‘hurt list’. This entitled them to receive ongoing treatment and, if the situation warranted it, half pay until they were fit to return to duty. Dockworkers who were more generally unwell and suffering from an injury or ailment not attributable to the dockyard were designated as ‘sick’ and entered on the ‘sick list’. These workers were not entitled to either free healthcare or half pay. However, reporting to the dockyard surgery for examination was the mechanism by which they could safeguard their jobs until they were fit enough to return to work. Particularly after 1859, when dockworkers were included in the Civil Service Superannuation Scheme,^34^ it also had wider implications in terms of demonstrating continuous service. Thus, where there were royal dockyards, a sizeable proportion of the local population was eligible for free healthcare, delivered via state-run medical facilities. Moreover, as we shall see, this was provided on a systematic basis, rather than being delivered in the *ad hoc*, inadequate and often unpredictable manner which characterised the schemes of commercial employers at this time, such as colliers and railway companies.^35^ These points are rarely appreciated in the secondary literature and historians have yet to fully explore their significance in relation to the broader development of healthcare provision in naval dockyard towns.^36^

In addition to dockworkers, various other groups connected with the dockyards formed part of the surgeon’s client base, such as the dockyard police. Men employed by contractors involved in building works at the various yards also seem to have ended up at the surgery when they were injured. At Portsmouth Dockyard, the surgeon also looked after the officers, their families and their servants, who were attached to the Royal Naval College, the School of Naval Architecture (closed 1832) and the Central School of Mathematics and Naval Construction.^37^ These establishments were located in the dockyard and at any given time could have between 400 and 500 residents.^38^ This particular group were entitled to comprehensive healthcare. Correspondence between the surgeon and the Victualling Board in 1827 (which at the time had responsibility for the medical affairs of the Navy) indicates that professional attendance was expected in all cases except midwifery.^39^

**Graph 1:**
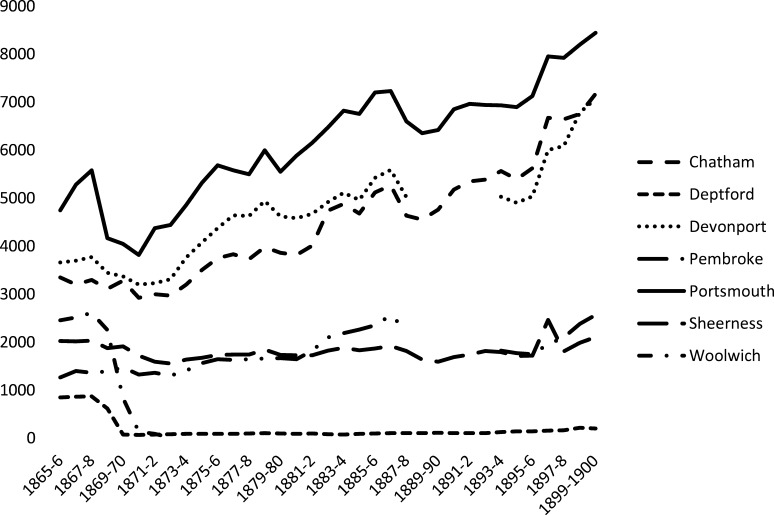
Average number of dockworkers eligible for healthcare, 1865–1900. Source: NA ADM49/181, Numbers of Workmen in Royal Naval Dockyards 1805–1900. Note: Deptford and Woolwich Dockyards closed in 1869.

Graph 1 shows the average number of dockworkers who were eligible for healthcare from 1865 onwards. Despite there being limits to what was officially supposed to be treated, this reveals that in numerical terms the population served by dockyard surgeons was substantial.

It is difficult to be precise about what this equated to in terms of an actual caseload. Part of the problem is a lack of sources; it appears that the only sick and hurt lists to have survived are from Portsmouth, with the first covering the years 1810–15 and the second, 1873–7.^40^ In addition, there is evidence to suggest that year-on-year in any given yard, the numbers of hurt cases (serious and slight) were at least partly determined by factors such as the nature of work being undertaken.^41^ After 1877, figures relating to sick and hurt cases in the dockyards start to appear in the annually published Statistical Report of the Health of the Navy.^42^ However, with the exception of the years 1880–3, these only report serious hurts which, as is evident from other sources, comprised just a small fraction of a dockyard surgeon’s caseload.

Some of the best data available relates to the period 1861–6. This comes from Portsmouth, where, amongst the medical department’s correspondence files, there is a table constructed by the surgeon in charge providing statistical detail on hurt cases for these years.^43^ Figures taken from this are reproduced in Table 1. Clearly, there is much that can be done with such data. From the percentages calculated in the last column it appears that serious hurts were on the increase. In other research with which the author is engaged, this type of analysis is being conducted. One aim is to assess how long-term patterns of injuries in the yards were affected by factors such as the introduction of new machinery and materials. Early findings from this work have revealed that hernias, along with contusions and fractures, were common throughout the century and affected all occupations in the yards. After the introduction of iron in the 1860s, burns (especially to the hands) and injuries to the eyes became more prominent.^44^ For the purposes of the current discussion, however, the key point to take away from Table 1 is that dockyard surgeons and their medical departments were very busy. In a six-year period a total of 13,140 new cases of hurt were treated, many of which would have required ongoing care. Furthermore, these figures do not include sick cases. Although requiring more detailed analysis, data extracted from a small sample of the Statistical Reports of the Health of the Navy suggest that for every new case of serious hurt there were roughly two to three cases of sickness recorded.^45^

**Table 1:** Total hurts recorded at Portsmouth Dockyard, 1861–6.

Year	Workforce	Slight hurts not requiring to be put on the list	Serious hurts requiring to be put on the list	Total hurts	Serious hurts as a percentage of total hurts
1861	4314	1560	317	1877	16.9%
1862	3303	1680	364	2044	17.8%
1863	3303	1700	371	2071	17.9%
1864	3296	1760	451	2211	20.4%
1865	3245	1830	566	2396	23.6%
1866	5400	1910	631	2541	24.8%
Total		10,440	2700	13,140	

## Everyday Duties and Responsibilities

The everyday activities undertaken at dockyard surgeries remained more or less unchanged throughout the period. They functioned as a cross between what we would today associate with a general practice and a hospital’s accident and emergency facility: medical examinations were undertaken; drugs, bandages and the like were dispensed; wounds were dressed; injuries and minor ailments were treated. By the mid-1860s minor surgical procedures were also being performed with some frequency. In June 1867, Dr Cree, whom we met earlier, wrote to the medical director general of the Navy asking to be supplied with one of Dr Richardson’s anaesthetic sprays, as he felt this would be of ‘great service’ in relieving pain and facilitating a number of ‘minor and painful operations’ that he was required to undertake.^46^ By 1878, it is known that amputations were being performed at Sheerness Dockyard.^47^ Such evidence points not just to the dangerous nature of naval shipbuilding but also says something about dockyard surgeons. Cree’s request for Dr Richardson’s spray came within a year of its invention.^48^ If Cree was as typical as he appears, then a picture begins to emerge of a group of practitioners who were up to date with developments in medical science and were prepared to embrace change where they perceived it would benefit medical practice. By this time, an empirical and experimental approach to medicine was already well established in the Royal Navy.^49^ It is therefore not surprising to find the same ethos in evidence at the royal dockyards, given that their medical officers had come from the Navy.

Surgeons treated men with hurts as and when the need arose. This was usually at the surgery itself but, if circumstances dictated, then the surgeon would go to the patient – wherever he happened to be in the yard at the time. Sometimes, in the case of very serious injuries, this might result in the patient being referred to a naval hospital. As a very rough guide, approximately one in every sixty serious hurt cases ended up in hospital.^50^ Access to these hospitals was a great benefit to dockworkers. They not only offered nursing care and medical treatment, but also, from 1852, used chloroform in surgical procedures.^51^ This was just five years after its very first use as a general anaesthetic by the Edinburgh obstetrician James Young Simpson. It was also a year before it was used to aid the delivery of Prince Leopold, Queen Victoria’s eighth child. This event is frequently regarded as a watershed moment in the lead-up to its widespread use.^52^ In other words, the Navy were early adopters of this advance, with dockworkers benefiting accordingly.

Men who were sick were also regular visitors to the dockyard surgery. They were assigned a lower priority than men with hurts. With the exception of those who were too ill to attend, patients on the sick list or wishing to be entered on it were expected to report to the surgery usually at a set time each day.^53^ These men were not supposed to receive any treatment. This clinic was all about establishing, through medical examination, whether a man was genuinely unfit for duty. Parallels can be drawn here with the armed services, where the detection of malingering became a priority in the nineteenth century.^54^ At the dockyards, concern that men might be faking or exaggerating injuries and illnesses is a recurrent theme in the records. As we shall see, the growing importance attached to this aspect of the surgeon’s role created tension in his relationship with the workforce. Like his counterparts in the army and Royal Navy, the dockyard surgeon’s duties and responsibilities increasingly went beyond just the treatment of patients.

Despite the official reasons for the daily ‘sick clinic’, there is, however, evidence that free treatment was nonetheless given to men designated as sick. How often this happened is difficult to say. As the practice contravened laid-down policy, it was not something that was readily recorded by dockyard surgeons. Whether treatment was given almost certainly depended as well on a process of negotiation between surgeon and patient, mediated by outside factors such as how busy the clinic was at the time. The impression gained from comparing the correspondence files at Portsmouth and Chatham which have survived from the 1850s and 1860s, with a detailed surgeon’s casebook from Sheerness covering the period 1867 to 1896, is that the treatment of men on the sick list occurred more often in the latter two decades of the nineteenth century. Broadly speaking, this fits chronologically with the wider official recognition of industrial diseases.^55^ For dockyard surgeons, the spectre of industrial disease muddied the previously clear distinction that they were able to draw between sick and hurt. This was particularly the case with lead poisoning. From the late 1860s, surgeons began to recognise this disease as attributable to the dockyard. With increasing regularity, cases which were previously classified as ‘sick’ were recorded as ‘hurts’. This of course gave patients an entitlement to free healthcare and half pay.^56^

Widow Biddlecombe’s petition for a pension following the death of her husband in 1849 is one of the few explicit examples from the first half of the century of a sick case receiving treatment. In a defensively worded letter to the admiral superintendent of Portsmouth Dockyard, the surgeon was keen to establish that he and his assistant had gone beyond the call of duty in their dealing with this patient. Biddlecombe, he advised, ‘was not a surgical case’, and as such ‘it was not my *duty* [original emphasis] to attend upon him’. In the surgeon’s opinion, Biddlecombe was not eligible for treatment; he had died not because of his work in the dockyard, but because he was elderly and suffering from bronchitis. Yet, despite this assessment, the surgeon confirmed that both he and his assistant had still ‘often’ attended the patient in his home prior to death.^57^

In addition to seeing patients at the dockyard surgery, the surgeon or his assistant also visited patients in their homes. Without doubt this daily round was the surgeon’s most onerous duty. In September 1847, James Henderson, the surgeon in charge at Portsmouth, wrote to Sir William Burnett (Director-General of the Medical Department of the Navy) asking for a second assistant surgeon to be appointed. Using a précis of an ‘average day’ to justify his request, he complained that the daily round had become a full-time occupation for one person. This meant that the other medical officer was a ‘constant prisoner’ of the surgery, where he did his best to manage the flow of patients and all the other duties associated with its running.^58^ These sentiments were echoed sixteen years later by Staff Surgeon William Gunn at Chatham. The daily round he commented, ‘is a work of considerable labour as the neighbourhood is very extensive, and the men’s residences often situated in nearly opposite directions, miles apart’.^59^

The twofold function of this daily round helps to illustrate the ambiguous nature of the surgeon’s role. On the one hand, the round ensured that men on the hurt list received ongoing treatment. At a time when seeing the doctor usually cost money, the dockyard surgeon was a welcome sight and the free healthcare he provided was a considerable benefit. The level of care given was by no means cursory either. If a man was ill enough (injured or sick), then he could expect to be visited periodically throughout the day. On the other hand, the round was also about surveillance or the ‘policing of health’. Under the article of instructions number 4, the sick were supposed to be visited each day in order to: ‘ascertain that the men are really ill and not absenting themselves for private purposes’.^60^ The usual consequences of being found guilty were instant dismissal. Hence a man’s livelihood could quite literally rest on the surgeon’s words. Sometimes the evidence against a man was incontestable, such as in the case of William Tiltman, a sawyer from Chatham Dockyard. On making an unannounced visit to the patient (who had been reporting in sick for a month), the surgeon found:

In the front part of his residence there is a Butcher’s shop marked with his name – In passing through this shop I found Tiltman in a room behind with a Butcher’s dress on, smoking a pipe – He had all the appearance of having been very recently employed in killing or cutting meat.^61^

But, when dismissal occurred based purely on a medical assessment, there was always the chance that the surgeon might mistake a genuinely sick man for a malingerer. This possibility caused tension. In extreme cases, surgeons were even threatened by men who felt that they had been responsible for getting them dismissed.^62^

Unsurprisingly, surgeons seem to have been only too well aware of how this aspect of their role adversely affected their relationship with the workforce. As a result, a practical approach to the problem seems to have been adopted by some surgeons, with not every case that came to their attention being reported up the line. In his memoirs Surgeon Rear-Admiral T.T. Jeans, who served at Pembroke Dockyard right at the end of the nineteenth century, fondly remembered how:

As part of my duty, I had to ride round the country and visit dockyardmen who had been reported absent ‘sick’, and during the spring and at other times – especially ‘potato time’ – it was often amusing, as I rode up a lane towards a cottage, to see, over the hedge, the poor ‘sick man’ busy hoeing his ground. He would hear the horse’s hoof, look up, catch sight of me and dash for his cottage and his bed, where after listening to a long-winded account of his ailments from his wife and hearing thumps on the floor overhead, I would find him probably fully dressed but minus his boots.^63^

As might be gleaned from the discussion so far, dockyard surgeons also had an important role supervising the work of their assistants. In larger yards this responsibility extended to the medical department’s messenger(s). Although surviving sources tell us much about daily life in the surgery, unfortunately they say less about the relationship between surgeons and their staff. Nor do they offer much in the way of explicit evidence about the way surgeons chose to delegate and apportion their department’s daily workload. Mutual respect for assistants as fellow medical practitioners certainly existed. When Dr Gunn retired from Chatham in 1865 he made a point of praising his assistant, Dr Holman. In what was his final entry in the department’s records, Gunn commented that Dr Holman was a man of great ’professional skill and ability’ who, at all times, discharged his duties with ‘zeal and energy’.^64^ Successive surgeons at Sheerness, Portsmouth and Chatham also called upon their assistants to provide second opinions in difficult or complex cases. Similarly, many of the departments’ everyday duties seem to have been shared. As we have already seen, turns were taken doing the daily round and running the surgery. Messengers also had a more expansive role than one might have expected, given that they were employed to ‘convey messages to various parts of the yard’.^65^ At Chatham, there is evidence of them manning the surgery when all the medical officers had been called away. This included providing first aid to patients on their arrival.^66^

Notwithstanding the above, the professional regard one would expect between naval officers of differing ranks was apparent too, as was the surgeon’s greater prominence in some areas of his department’s activities. As the senior officer, he appears to have had more involvement than his subordinate(s) with patients who had sustained very serious injuries, particularly those where hospital admission was required. The assessment of long-term sick and hurt cases, and paying unannounced visits to suspected malingerers, were also duties that seem to have been undertaken almost exclusively by the surgeon. This suggests that assistants took on more of the mundane work. Such a conclusion does, however, warrant a degree of caution. Naval protocol dictated that all correspondence was signed by the medical officer in charge and so the skewing effect this has had on the records needs to be taken into account.

To complete this outline of the everyday duties of dockyard surgeons, it is also possible to say something about the hours they kept. As the prioritisation of patients and the eligibility criteria for free healthcare indicate, the primary function of dockyard medical departments was to provide treatment to employees who were injured while at work. By its very nature, the dockyard was a place where medical emergencies could occur both day and night. Contractors, for instance, often continued to work irrespective of normal dockyard hours. Hence, although dockyard surgeries kept regular hours, the surgeons themselves had to be available beyond this. At Portsmouth, where the client base included people not directly connected with the yard, surgeons were effectively on call twenty-four hours a day. In December 1851, for example, the surgeon was called to attend Edward James Reed, a student of the Mathematical School, whom he found labouring ‘under great excitement’ in a ‘delirious state’ and being ‘very noisy’. After administering an anodyne, the surgeon remained with the patient until midnight, when eventually he calmed down and fell asleep.^67^

## Other Duties and Responsibilities

In addition to ensuring the day-to-day smooth running of their medical departments, dockyard surgeons had a number of other duties and responsibilities. Although not an official requirement of the post, the building of links with fellow practitioners at nearby naval establishments was a vital task. The importance of this is best illustrated by looking at an example of what could happen when such links had not yet been established. Less than a month after his appointment to Portsmouth Dockyard, and probably full of enthusiasm for his new post, Dr Cree decided it would be a good idea to review the long outstanding cases on the hurt list. In doing so, he identified two patients whose treatment and recovery he believed was being hindered by their domestic living conditions. He therefore arranged for them to be sent to Haslar Hospital. However, on arrival, both were refused admission and promptly returned, bearing a curt notification ‘that such cases are not received from the dockyards’.^68^ This was not a good start for Cree. Aside from the potentially negative impression he had created with his colleagues at Haslar, he had the embarrassment of reporting the matter in only his second written communication with the admiral superintendent.

Establishing a rapport with civilian practitioners was similarly important.^69^ Collaboration in the form of joint consultations and written dialogue took place on a fairly regular basis, and could be particularly useful in dealing with difficult cases. The example of William Cole, a wood turner at Sheerness Dockyard, is typical. After having put Cole on the sick list, Fleet Surgeon Hoggan conferred with the patient’s private doctor, who agreed with his diagnosis of phthisis. The two men then liaised over the patient’s ongoing treatment and care, concurring several months down the line that there had been little or no progress made, nor was there ever likely to be. As a result, Hoggan recommended Cole’s discharge from the yard as an invalid.^70^ Co-operation of this nature thus provided reassurance for dockyard surgeons and was a way of pooling expertise. Knowledge that a sick dockworker was being attended by a private practitioner also helped to allay fears of malingering, which in turn reduced the need for home visits. Other practical benefits were derived from having a good appreciation of the local medical market. When James Henderson, the surgeon at Portsmouth, needed to recruit a new messenger he was quick to use such intelligence, recommending Thomas Hayland for the post. Hayland, he advised the admiral superintendent, was ‘well qualified to fill the situation’, having been ‘brought up in, and now employed at a chemist and druggist’s shop … having at one period been attached to an hospital’.^71^

Many of the surgeon’s remaining duties were administrative in nature. On an annual basis they were required to prepare their department’s financial accounts for external auditing. At the same time they also had to submit a bulk order for medicine and medical stores, sufficient to cover their dockyard’s ordinary needs for a twelve-month period. The surgeon in charge at each dockyard was also responsible for accurately maintaining the sick and hurt lists. Although few of these seem to have survived, it is clear from other sources that they were vital documents, regularly examined by dockyard superintendents. The Medical Department of the Navy also always requested copies as part of their annual visit and inspection of dockyard medical departments. The importance of these lists is easily explained. Not only did they provide up-to-date information on available manpower but also, as we have already seen, they were used to determine entitlements to healthcare and half pay.

As the nineteenth century progressed, the administrative burden on dockyard surgeons grew. With naval spending often in the spotlight, increased central demands for information required surgeons to keep ever more comprehensive records of their activities. The establishment of the Medical Department of the Navy in 1833 seems to have done little to stifle the developing appetite for information.^72^ By 1847, in addition to the reports already mentioned, surgeons in charge of dockyard medical departments were expected to provide: a quarterly nosological, medical and surgical report; an annual report of the receipt and expenditure of medicines and stores; an annual return of the issue of rupture trusses, accompanied by a receipt from each of the recipients. Further orders were received in November 1851, instructing all surgeons and their assistants to also keep a journal, documenting their activities in the yard.^73^

Increased emphasis also started to be put on the keeping of detailed patient case notes (over and above the traditional sick and hurt lists). The Lord High Admiral first issued orders to this effect in 1827, instructing dockyard surgeons and their assistants to keep a ‘sick book’ for inspection by the medical commissioners of the Victualling Board. This was to contain ‘a full and detailed history of all the most important and interesting cases falling under their management’.^74^ So far, it appears that the only such books to have survived are those from Portsmouth and Sheerness, the earliest dating from 1866. If the case of Portsmouth is anything to go by, then it seems that the order to keep such books was initially greeted with little enthusiasm and to all intents and purposes forgotten, both by successive surgeons at Portsmouth and by the medical commissioners. Although the orders were reiterated in January 1833 this evidently changed little, with just two cases being recorded in Portsmouth’s sick book between 1827 and 1838. It was not until 1851, under the increasingly watchful eye of the Medical Department of the Navy, that an external inspection of Portsmouth brought to light the fact that the order had not been satisfactorily implemented.^75^ In his apology to Sir William Burnett, the surgeon acknowledged that he had ‘not perhaps carried out the spirit of the said order’ and confirmed that from now on the matter would receive his ‘particular attention’.^76^

How, then, can this steady bureaucratisation of dockyard medical departments be explained? And what does it tell us about the changing role of dockyard surgeons and the healthcare that was on offer to dockworkers? It is tempting to see the increase in red tape as just symptomatic of reforms made by successive Boards of the Admiralty aimed at achieving greater efficiency in the royal dockyards. These began with the appointment of Sir James Graham as First Lord of the Admiralty in November 1830. Graham was a Whig politician determined to reduce spending, and quickly set about overhauling the administration of the Navy, beginning with the abolition of the Navy Board in 1832.^77^ Red tape was an issue to some extent  and without doubt much of the information that surgeons were increasingly required to keep helped to establish an audit trail and exposed their departments to external scrutiny. Yet this was only one of the reasons for the increase in bureaucracy and arguably not the most important.

Of greater significance were the consequences that arose from the naval authorities’ decision to bear greater responsibility for the welfare of their civilian workforce. The first signs of this came in 1839 with the reintroduction of pensions for artificers and workmen on the establishment of the royal dockyards and victualling yards. Pensions had previously been paid to established men with thirty-five or more years’ service, but were abolished in 1832 for a seven-year period as part of Graham’s reforms.^78^ The 1839 scheme was much more generous than ever before. Those with twenty or more years’ service could now look forward to a pension when they became too old or too ill to work. Additionally, individuals could be superannuated (or in the event of death in service, their families compensated) in cases where their working life was interrupted or ended prematurely as a consequence of injury or ill health attributable to the dockyard.^79^ Further improvements occurred in 1859, when the arrangements in operation at the royal dockyards were incorporated into the wider Civil Service Superannuation Scheme. This saw a relaxation of the eligibility criteria for pensions and a substantial increase in the benefits paid (even labourers now qualified for a pension of up to £31 6s p.a.).^80^

Clearly, there were financial implications attached to providing such a scheme. Moreover, the superannuation of employees whose working life was ended prematurely as a result of ill health was an aspect of the new arrangements that was potentially open to abuse from spurious claims. The most important effect that this had on dockyard surgeons was that they were increasingly called upon to act as expert witnesses in petitions for compensation or where superannuation was claimed on the grounds of ill health. Although they had always performed such a function in respect of the sick and hurt lists and under the old pension arrangements, this aspect of their role now became greatly expanded.^81^ In effect, by extending their responsibility as employers, the authorities created a new dependency on dockyard surgeons. This is very evident in the records, not just in terms of the frequency with which medical reports associated with superannuation claims appear, but also in the general prominence given to the matter. A case from Chatham illustrates this well. In November 1861, the surgeon received a tip-off that the shipwright Edward Dalton, who was currently on the sick list, had been seen in the ‘Siren’s Head’ in an intoxicated state. As a result, the surgeon decided that the time had come to raise his concerns about this man with the captain superintendent. In his report, he commented:

[Dalton] has been complaining for very many months of symptoms of Rheumatism which, he says, prevents him following his employment as a shipwright, but as his general health is good, and there seems no evidence of his suffering in the way he states, I have every reason to believe that he is malingering.

He went on to add:

Every possible indulgence has been granted to him for nearly fifteen months, and he has had leave twice to visit Ireland his native country. On returning lately to Chatham though apparently in excellent health he did not go to work, and still persists in saying that he is unable to do so….

It is with great reluctance that I make this complaint, but as Dalton’s conduct may, by setting a bad example, have an injurious effect upon his fellow workmen I consider it my duty to do so.^82^

Shortly after this report, Dalton was called before the captain superintendent where he admitted the incident at the Siren’s Head and was promptly dismissed for drunkenness while on the sick list. The matter did not, however, end here. Indeed, it is the surgeon’s comments that followed Dalton’s subsequent request for a pension that are most revealing. He remarked:

I am still of the same opinion, that Dalton had been feigning illness for some considerable time with the hope of getting his discharge from HM’s service with a pension…. Dalton could not be recommended for superannuation as he wished, being only 48 years of age, as he could not be given the medical certificate necessary by the 19th section of the superannuation act of April 1859, to satisfy the commissioners of the Treasury.

It is rather singular that this man’s complaints began immediately after the passing of that Act of Parliament which he fancied entitled him to a pension though he had only then served eighteen years in H.M. Service. In less than nineteen months he has been 543 days on the sick list….

I may also mention that Dalton is a bachelor, and that it is well known that he disposed of some property in this neighbourhood a short time since with the intention, it is supposed, of retiring to Ireland when he obtained his pension.^83^

These comments reveal not just the sharper edge that the 1859 Superannuation Act brought to this aspect of the surgeon’s role, but also help us to appreciate the complicated nature of the relationship that surgeons had with the workforce. Their responsibilities now included policing a system of health and welfare benefits and, as the surgeon in the above letter appeared acutely aware, like any other system, it could be worked, cheated and manipulated.

In order to fulfil this rapidly developing aspect of their role, it became necessary for surgeons to maintain ever more accurate and comprehensive notes on their patients. Under the 1839 arrangements, the superintendents of the various dockyards were required to provide the Admiralty with a completed pro-forma for each of those men applying, or being recommended, for superannuation. This was designed to assist the decision-making process and was used to determine the level at which any pension or compensation was paid. The pro-forma included an assessment of the applicant’s moral character and, crucially, for dockyard surgeons, a section in which to record full details of ‘the disease or hurt which may render a man unfit for further service … stating whether the disease or injury has been occasioned by the service’.^84^ It is interesting to note that the introduction of this pro-forma roughly coincided with the promise by the surgeon at Portsmouth to pay ‘particular attention’ to the recording of cases in the ‘sick book’. This was presumably because he could now see the practical value of such a record, as it made the completion of superannuation forms that much easier.

It is not clear how long this pro-forma remained in use, but the indications are that the medical section of it had become obsolete by the 1850s. By this date correspondence files from Portsmouth Dockyard show that the naval authorities and the admiral superintendent of the yard expected full medical reports in support of claims. Similarly, when one of the medical officers proactively recommended a person for superannuation, he began to pre-empt such requests by providing medical reports at the outset, as a matter of course. The report prepared in 1865 concerning Mr Grant Smith illustrates the level of detail that these could contain:

In accordance with your directions to examine and report on the health and physical condition of Mr Grant Smith I beg to state in 1856 he received a severe contusion of the back and loins from a fall off a ladder while he was employed in the Dockyard Fire Brigade … which injury kept him in the Haslar Hospital for 7 weeks when he returned to light duty … he has continually suffered since this from pain at the parts referred to which prevents him lifting heavy weights or using much exertion … in March last year he was placed on the sick list for a severe rheumatic attack… which prevented him doing duty for upwards of 3 months since which time he has been employed in light duty … he complains of frequent attacks of giddiness … therefore taking into consideration his previous history and present condition I would beg to recommend him for superannuation.^85^

It is also pertinent to observe that the patient history contained in this report covered a nine-year period, during which time two different people had been in charge of the dockyard’s medical department. The report was therefore not written from memory. At the time of its submission, the author had only been in post for approximately five months.^86^ This confirms that by the 1850s, patient notes of sufficient detail were being kept to enable thorough case histories to be constructed, even when the surgeon and the patient had only limited contact. The extent to which these notes were put to any therapeutic or medical use is very difficult to say. The surviving volume from Sheerness which was mentioned earlier does, however, contain information that would be of just such value. Details of patients’ previous injuries and ailments along with notes on how they responded to certain treatments and drugs appear with many of the entries. That notes of this type were kept at all is significant within the broader history of patient records. Although the case books of individual doctors have been found as far back as the sixteenth century, current research suggests that the systematic collection of patient data was more a nineteenth-century phenomenon.^87^ So again, just as was noted earlier with chloroform, the Navy appear to have been relatively early adopters. Of course, such notes could have served a range of purposes. But the positive interpretation is that, as healthcare providers to a civilian workforce, the Navy were forward thinking when it came to implementing perceived advances in medical practice.

This upbeat appraisal, which places dockyard surgeons at the forefront of developments in medicine, holds true when we look at their involvement in other areas. In October 1830, a circular originated by the Victualling Board (at the time responsible for medical affairs in the Navy), reveals both the board’s awareness that these officers were among the first to encounter and use new medical instruments, as well as a desire to derive wider benefit from their experiences:

A Book should be kept at the Hospitals and dockyards, showing the nature of any newly invented/ contrived instruments that may be received at those places for trial, the time they were sent, and whether any and what report has been made upon the same … you will also cause a quarterly report to be made to the Board.^88^

Periodically, the naval authorities also asked dockyard surgeons to conduct trials of medical appliances. Without exception, these were aimed at finding practical, ‘off-the-peg’ solutions to injuries commonly suffered by both dockworkers and sailors alike. Hernias received particular attention.^89^ The testing of Sale’s rupture trusses at Portsmouth in 1848–9 was just one of five similar trials that are known to have taken place during the first half of the century.^90^ In this particular case, because of patients complaining about the comfort of single trusses and springs breaking during the fitting of double trusses, the surgeon concluded that they possessed no advantage over those already in use.

Although such trials were centrally driven, they nonetheless gave rise to a two-way dialogue in which surgeons felt able to come up with their own ideas and solutions to the problems that they faced on a frequent basis. In June 1830, for example, the surgeon at Portsmouth received a consignment of airtight bandages for testing, which had been invented specifically for fractured limbs by his colleague Mr Cow at Woolwich Dockyard.^91^ A willingness to innovate and experiment can also be seen in the way that individual surgeons responded to the medical challenges thrown up by developments in naval shipbuilding. Eye injuries which, as was noted earlier, became more common following the introduction of iron, provide an excellent example. At Portsmouth, Dr Cree took a conventional approach to the problem, requesting an ophthalmoscope to aid his examinations.^92^ At Chatham, however, the surgeon responded very differently, asking to be supplied with a magnet.^93^ He evidently felt that this would be an effective way to remove the particles of iron, which were a prominent feature of many eye injuries. The use of magnets in this way was not unheard of at the time; a single report of an experiment using them had appeared in *The Lancet* some years previously.^94^ However, their use in eye surgery did not develop until the last decades of the nineteenth century and only started to gain prominence in the Edwardian period.^95^ Although it is not known whether magnets were eventually used in the dockyards, the important point is that the surgeon felt he was working in an environment where it was completely acceptable to suggest trying new and novel techniques.

These, and similar items of correspondence, reinforce the argument that the Admiralty’s need to maintain a healthy workforce encouraged an experimental and empirical approach to medicine in the dockyards. The prevailing circumstances in the yards themselves were conducive to this as well. The huge numbers they employed provided surgeons with the necessary scope in terms of volumes of patients, while changing materials and technology in naval shipbuilding presented them with new medical challenges.^96^ Similarly, although not quite the tightly controlled authoritarian communities of the Army and Navy,^97^ the dockyards’ civilian workforce was nonetheless highly ordered.^98^ Together with the healthcare on offer (which was free, comprehensive and delivered by experienced practitioners), this helped to make dockworkers a compliant patient group. It is also clear that, while the naval administration may have been bureaucratic, it nonetheless provided a mechanism through which surgeons could share best practice. Although more work needs to be done in this area, such conclusions fit well with Peter Mathias’ observations about medicine and the armed forces in the late eighteenth century.^99^ They also resonate with more recent research into the Sick and Hurt Board during the Seven Years War. This has drawn attention to the innovative and empirical scientific methodology that developed from medical trials aimed at addressing the problem of disease in the Western Squadron.^100^

The proactive ethos suggested by the above discussion can also be seen in dockyard surgeons’ final main area of activity. In addition to all their other duties and responsibilities, surgeons also performed sanitary and public health roles within the dockyards. Some of this was centrally led – both by yard superintendents and by the naval administration.^101^ The Admiralty, for instance, provided explicit instructions concerning the upkeep of official residences in the yards, which included whitewashing and cleaning in-between occupancies. Though the relevant circular was addressed to the superintendents of the various dockyards, the responsibility for ensuring that these aspects of the instructions were carried out was often delegated to the surgeon in charge.^102^ Superintendents also sought advice from the surgeon on issues ranging from rodent infestations to drainage and ventilation within the dockyard and its buildings.^103^

There is also plenty to suggest that surgeons also pursued their own preventative agendas while in post. In particular, surgeons were very keen to stop disease spreading amongst the workforce. As soon as risks were identified, prompt action followed. This can be illustrated by looking at what happened in March 1849, after it was discovered that a number of men belonging to different contractors in Portsmouth Dockyard were afflicted with smallpox. To contain the outbreak, the surgeon wrote to the admiral superintendent proposing the following course of action:

I beg leave to suggest for your consideration whether an order could be issued to prohibit the return of any person, who may be absent from small pox, without being examined at the surgery, and a certificate given of fitness for admission, whereby the spreading of the disease amongst the several workmen of the Dockyard will in some degree be checked.^104^

Of course, set within the broader context of naval medicine, it is no surprise to find that dockyard surgeons were on the ball when it came to disease containment. Having seen active service, they were no strangers to dealing with outbreaks of disease; at sea, naval surgeons faced the problem all too often.^105^ More specifically to smallpox, many naval surgeons were also involved in administering the Admiralty’s voluntary campaign of vaccination against the disease which was introduced during the Napoleonic Wars.^106^ Importantly, this example reveals how, at local level too, a proactive stance was adopted by surgeons towards dealing with health issues.

## Conclusion

Surgeons were, without doubt, very important figures in the royal dockyards. These highly experienced officers played a crucial role in looking after the health of dockworkers. Although referred to as surgeons, they nonetheless played the part of physician, dispenser and public health officer as well. The unique context of the dockyard encouraged innovation and allowed surgeons to embrace a more empirical and experimental approach to medicine. This often saw them at the forefront of medical developments. Regarded in this light, they can be seen as pioneers. Such a view does, however, need to be set against the complicated relationship that they held with patients. Their role as policeman and expert witness caused tension; their testimony could determine whether a man kept his job or received a pension.

Focusing on these practitioners also provides a way of illuminating and examining the naval authorities as healthcare providers to a civilian workforce. The manner in which the surgeon’s role steadily expanded testifies to the increasingly comprehensive provision that was on offer. In dockyard towns, literally thousands of people were eligible for a whole range of different benefits, including free healthcare, sick pay and old age pensions. Certainly by the last quarter of the century this amounted to something akin to a localised welfare state. Indeed, one might argue that the tension noted between surgeon and patient was an articulation of the rights and responsibilities that often characterise such systems of care. Of course, at the moment, conclusions of this nature can be no more than tentative; there is clearly much more work to be done. Perhaps most pressing is the need to understand how the royal dockyards interacted with other state, charitable and private healthcare providers in their vicinity. Thinking from the patient’s perspective, we might also ask whether it was better to be ill in a dockyard town. While these and many other questions still need to be answered, it seems clear that the royal dockyards warrant serious consideration in broader discussions about industrialisation and the development of systems of health and welfare provision.^107^

## Footnotes

1. National Archives, Kew (hereafter NA), ADM49/181, Numbers of Workmen in Royal Naval Dockyards 1805–1900. Although the numbers borne fluctuated on a yearly basis, the overall trend was upwards in the nineteenth century.

2. RileyR.C., ‘The industries of Portsmouth in the nineteenth century’, The Portsmouth Papers, 25 (1976), 3–22: 9; Penelope Corfield has noted that the dominance of the larger royal dockyards in local labour markets reached similar proportions in the eighteenth century, see P.J. Corfield, *The Impact of English Towns 1700–1800* (Oxford: Oxford University Press, 1982), 45.

3. The distinct social and economic characteristics of naval dockyard towns are discussed in G. Jackson, ‘Ports 1700–1840’, in P. Clarke (ed.), *The Cambridge Urban History of Britain 1540–1840*, 3 vols (Cambridge: Cambridge University Press, 2000), ii, 705–31: 720–1. The Admiralty’s policy of self-sufficiency in respect of the royal dockyards and their control over areas of shoreline also had a stifling effect on industry and commerce; see S. Palmer, ‘Ports’, in M. Daunton (ed.), *The Cambridge Urban History of Britain 1840–1950*, 3 vols (Cambridge: Cambridge University Press, 2000), iii, 133–50: 135. Many naval dockyard towns were also situated strategically for ease of defence and proximity to prospective enemies. As such they frequently lacked a coherent trading hinterland and foreland, which further hindered commercial development: R.C. Riley and J. Chapman, ‘Industrialisation in naval ports: the Portsmouth case’, in B.S. Hoyle and D.A. Pinder (eds), *Cityport Industrialisation and Regional Development: Spatial Analysis and Planning Strategies* (Oxford: Pergamon, 1981), 133–50: 133–4.

4. RileyRay, Portsmouth Ships, Dockyard & Town (Stroud: Tempus, 2005), 38

5. DickensCharles, The Uncommercial Traveller, Vol. 1875 (repr. Newcastle upon Tyne: Cambridge Scholars, 2008), 212

6. O’HaraGlen, Britain and the Sea since 1600 (Basingstoke: Palgrave Macmillan, 2010), 114–116

7. For examples, see D.K. Brown, *Warrior to Dreadnought: Warship Development 1860–1905* (London: Chatham Publishing, 1997); D.K. Brown, *Before the Ironclad: The Development of Ship Designs, Propulsion and Armament in the Royal Navy, 1815–60* (London: Conway Maritime, 1999); David Evans, *Building the Steam Navy: Dockyards, Technology and the Creation of the Victorian Battle Fleet 1830–1906* (London: Conway Maritime, 2004).

8. HaasJ.M., ‘Work and authority in the royal dockyards from the seventeenth century to 1870’, Proceedings of the American Philosophical Society, 124 (1980), 419–428; K. Lunn and A. Day (eds), *History of Work Labour Relations in the Royal Dockyards* (London: Mansell, 1999).

9. LloydChristopher and CoulterJack L.S., Medicine and the Navy, 1815–1900, 4 vols (London: E. & S. Livingstone, 1963), iv, 31.

10. The same cannot be said of commercial shipbuilding in the twentieth century. See, for example: R. Johnston and A. McIvor, ‘Dangerous work, hard men and broken bodies: Masculinity in the Clydeside heavy industries, c.1930–1970s’, *Labour History Review*, 69 (2004), 135–51; H. Murphy, ‘The health of electric arc welders and the adoption of arc welding in the British shipbuilding industry, 1930–1951’, *International Journal of Maritime History*, 17 (2005), 69–90.

11. CookGordon C., Disease in the Merchant Navy: A History of the Seaman’s Hospital Society (Oxford: Radcliffe Publishing, 2007), 302

12. MakepeaceMargaret, The East India Company’s London Workers: Management of the Warehouse Labourers, 1800–1858 (Woodbridge: Boydell Press, 2010), 86–87

13. WrightD.S., ‘The history and development of the medical services of H.M. Dockyard, Chatham, 1625–1966’, Journal of the Royal Naval Medical Service, 54 (1968), 25–684868312

14. WrightD.S., ‘Man-made mineral fibres: A historical note’, Journal of the Society of Occupational Medicine, 30 (1980), 138–140; D.S. Wright, ‘Training in occupational medicine in the Royal Navy – a personal view’, *Journal of the Royal Navy Medical Service*, 71 (1985), 9–14.7003259

15. BiddleRichard, ‘Naval shipbuilding and the health of dockworkers c.1815–1871’, Family & Community History, 12 (2009), 107–116; Richard Biddle, ‘Health and the royal dockyards’, *Dockyards*, 16 (2011), 10–11; Richard Biddle, ‘The health of workers in the royal dockyard, Portsmouth’, in R. Dunn and D. Leggett (eds), *Re-inventing the Ship* (Abingdon: Ashgate, 2012), 93–112.

16. Wright, *op. cit.* (note 13), 28.

17. WebbJ., QuailS., HaskellP. and RileyR.  (eds), The Spirit of Portsmouth: A History, (Chichester: Phillimore, 1997), 48

18. The *Navy Lists* make tracing provision across the dockyards an easy task.

19. For details of the qualifications required to become an assistant surgeon and a surgeon in the Royal Navy see Lloyd and Coulter, *op. cit.* (note 9), 20–5.

20. Between 1815 and 1899, 103 men served as head of medical department across the home yards. The careers of forty-six of these men were traced using the *Navy Lists* The sample comprised all those in post in 1815 and then every ten years thereafter until 1895. This revealed an average of twenty-six years’ experience as a naval surgeon at time of appointment, with no man having less than thirteen years. Twenty-two men in the sample were listed as having attained the higher degree of MD.

21. NA ADM 196/8, Admiralty: Officers’ Service Records (Series III), Medical Officers.

22. Cree’s service record can also be found in ADM 196. However, his journals provide a far greater insight into his career and experience prior to joining Portsmouth: M. Levien (ed.), *The Cree Journals: The Voyages of Edward H. Cree, Surgeon R.N. as Related in His Private Journals, 1837–1856* (Exeter: Webb & Bower, 1981).

23. HaycockD.B. and ArcherS.  (eds), Health and Medicine at Sea 1700–1900, (Woodbridge: Boydell Press, 2009).

24. Lloyd and Coulter, *op. cit.* (note 9), 20.

25. *New Navy List*, January 1847.

26. NA ADM 181/70, Navy Board and Admiralty: Navy Estimates, 1868–9.

27. The medical market was highly competitive in the nineteenth century and many doctors struggled to establish viable private practices. See Anne Digby, *Making a Medical Living: Doctors and Patients in the English Market For Medicine, 1720–1911* (Cambridge: Cambridge University Press, 1994).

28. CookGordon C., ‘Richard Dobson Kt MRCS FRS (1773–1847) and the inferior status of naval medicine in the early nineteenth century: End of the fleet physicians’, Notes & Records of the Royal Society, 59 (2005), 35–431573632210.1098/rsnr.2004.0071

29. *Navy Lists*, 1880–1900.

30. Chatham Royal Dockyard Library (hereafter CRDL), 2011-0229 Doc 1182 Letter Book Surgery at Chatham August 1859–July 1871, Letter 7, July 1865.

31. National Maritime Museum (hereafter NMM), POR/J/6, One Volume of Orders to the Yard Surgeon, 1823–49, copy of order dated 25 April 1815.

32. Haas, *op. cit.* (note 8), 421.

33. Prior to 1806 the ‘Sick and Hurt Board’ was responsible for the health of seamen: Patricia K. Crimmin, ‘The Sick and Hurt Board and the health of seamen c.1700–1806’, *Journal for Maritime Research* (December 1999), 1–17.

34. FieldJohn, ‘Portsmouth Dockyard and its workers 1815–75’, The Portsmouth Papers, 64 (1994), 3–22: 12.

35. BronsteinJamie L., Caught in the Machinery: Workplace Accidents and Injured Workers in Nineteenth Century Britain (Stanford, CA: Stanford University Press, 2008), 32–58

36. In Portsmouth, numbers of private practitioners in the vicinity of the dockyard steadily declined as workforce numbers grew, see: Richard Biddle, ‘Dissecting the medical marketplace: The development of healthcare provision in nineteenth century Portsmouth’ (unpublished PhD thesis, Oxford Brookes University, 2009).

37. NMM POR/J/6, letter 15 March 1827.

38. Royal Naval Museum (hereafter RNM), 1983/621–2, 12/1–3. The Records of the Medical Department, HM Dockyard Portsmouth, 1810–15, 1850–1955, letter 29 July 1865.

39. NMM POR/J/6, various items of correspondence March 1827.

40. RNM 1983/621–2: 2/1–11 Hurt Books, 1873–7; 3/1–3 Medical Absence Registers, 1810–15.

41. Biddle, ‘The Health of Workers’, *op. cit.* (note 15), 102–12.

42. The first of these parliamentary reports was published in 1840. For their in-depth analysis, see Vaughan M. Dutton, ‘Mortality decline in the Royal Navy, 1850–99’ (unpublished DPhil thesis, University of Oxford, 2009).

43. RNM 1983/621–2, 12/1–3, Statement of the Number of Hurts Accrued 1861–6.

44. For a more detailed discussion, see Biddle, ‘The Health of Workers’, *op. cit.* (note 15), 102–12.

45. The royal dockyards feature in the reports from 1877. One report every five years was examined (1881, 1885, 1889, 1893 and 1897).

46. RNM 1983/621–2, 12/1–3, letter 20 June 1867.

47. NA ADM 105/79, Register of Important Cases Found in RN Dockyard, Sheerness, 1867–96, entry dated 14 March 1878.

48. For further details, see http://www.sciencemuseum.org.uk/objects_old/anaesthesiology/A602928.aspx[accessed 20 April 2012].

49. MathiasPeter, ‘Swords and ploughshares: The armed forces, medicine and public health in the late eighteenth century’, in P. Mathias (ed.), The Transformation of England: Essays in the Economic and Social History of England in the Eighteenth Century (London: Methuen, 1979), 265–287; 268–9.

50. Biddle, ‘Naval shipbuilding’, *op. cit.* (note 15), 113.

51. Lloyd and Coulter, *op. cit.* (note 9), 48.

52. PorterRoy, The Greatest Benefit to Mankind: A Medical History of Humanity From Antiquity to the Present (London: Fontana Press, 1999), 367–368

53. NMM POR/J/6, general order 26 June 1848.

54. CooterRoger, ‘Medicine in war’, in D. Brunton (ed.), Medicine Transformed: Health, Disease and Society in Europe, 1800–1930 (Manchester: Manchester University Press, 2004), 331–363; 351–2.

55. BartripPeter W.J., The Home Office and the Dangerous Trades: Regulating Occupational Disease in Victorian and Edwardian Britain (Amsterdam: Rodopi, 2002), 7–1112740161

56. For more, see Biddle, ‘The Health of Workers’, *op. cit.* (note 15).

57. NMM POR/J/6, letter 8 March 1849.

58. NMM POR/J/6, letter 29 September 1847.

59. CRDL 2011-0229 Doc 1182, letter 9 May 1863.

60. NMM POR/J/6, letter 29 September 1847.

61. CRDL 2011-0229 Doc 1182, report on Wm Tiltman, Sawyer, 5 October 1864.

62. CRDL 2011-0229 Doc 1182, pencil annotation added to letter to Admiralty 21 November 1860.

63. JeansT.T., Reminiscences of a Naval Surgeon (London: Sampson Low Marston & Co., 1927), 126–127

64. CRDL 2011-0229 Doc 1182, comments, 3 September 1865.

65. RNM 1983/621–2, 12/1–3, letter 9 October 1866.

66. CRDL 2011-0229 Doc 1182, report 10 October 1863.

67. RNM 1983/621–2, 12/1–3, letter 8 December 1851.

68. RNM 1983/621–2, 12/1–3, letter 13 October 1864.

69. The importance of professional networks to success in private practice has long been recognised. Interaction between military/naval practitioners and civilian practitioners in this context is an area yet to be fully explored by historians: Anne Digby, *The Evolution of British General Practice 1850–1948* (Oxford: Oxford University Press, 1999), 95–100.

70. NA ADM 105/79, report 4 August 1879, with annotation dated 6 September 1879.

71. RNM 1983/621–2, 12/1–3, letter 19 May 1854.

72. Between 1806 and 1832 the Navy had no distinct medical department. Instead responsibility for medical affairs came under the Transport Board in 1806–17 and then, following its abolition, the Victualling Board. The situation was rectified following the reforms made by Lord Graham. These established the ‘Physician of the Navy’ as one of the five Principle Officers of the Admiralty. By 1843, the title had changed to ‘Director-General of the Medical Department of the Navy’. See Lloyd and Coulter, *op. cit.* (note 9), 2.

73. RNM 1983/621–2, 12/1–3, order 26 November 1851.

74. NMM POR/J/6, order 18 July 1827.

75. RNM 1983/621–2, 12/1–3, letter 8 December 1851.

76. *Ibid*

77. RodgerN.A.M., The Admiralty (Lavenham: Dalton, 1979), 98–104

78. Field, *op. cit.* (note 34), 12.

79. NMM POR/J/6, copy of letter 14 May 1841, sent by the Admiralty to the admiral superintendents of each dockyard.

80. Field, *op. cit.* (note 34), 12.

81. As others have noted, the doctor as an expert witness was a facet of medical practice which expanded during the nineteenth century. This occurred, for example, in criminal trials and in providing evidence to various parliamentary investigations. See Bartrip, *op. cit.* (note 55); R. Smith, *Trial by Medicine: Insanity and Responsibility in Victorian Trials* (Edinburgh: Edinburgh University Press, 1981); Katherine D. Watson, *Forensic Medicine in Western Society* (Abingdon: Routledge, 2011).

82. CRDL 2011-0229 Doc 1182, report 21 November 1860.

83. CRDL 2011-0229 Doc 1182, report to Lords of Treasury 15 February 1861.

84. NMM POR/J/6, copy of letter 14 May 1841, *op. cit.* (note 79).

85. RNM 1983/621–2, 12/1–3, medical report on Mr Grant Smith, 1865 (thought to be March).

86. *Navy List*, January 1866.

87. LaneJoan, The Making of the English Patient: A Guide to Sources for the Social History of Medicine (Stroud: Sutton, 2000), 92–116 For a comparison between early case books kept by individual doctors and the systematic recording by nineteenth-century institutional healthcare providers, see: S.T. Anning, ‘A medical case book: Leeds 1781–1784’, *Medical History*, 28 (1984), 420–31; Jonathan Andrews, ‘Case notes, case histories, and the patient’s experience of insanity at Gartnard Royal Asylum, Glasgow in the nineteenth century’, *Social History of Medicine*, 11 (1998), 255–81.10.1093/shm/11.2.25511620430

88. NMM POR/J/6, circular 25 October 1830.

89. RodgerN.A.M., The Wooden World: An Anatomy of the Georgian Navy (London: HarperCollins, 1988), 103

90. NMM POR/J/6, letter reporting on trial of Sale’s rupture trusses, 6 January 1849.

91. NMM POR/J/6, letter 16 June 1830.

92. RNM 1983/621–2, 12/1–3, letter 11 July 1868.

93. CRDL 2011-0229 Doc 1182, letter 25 July 1865.

94. *The Lancet*, 15 October 1859.

95. ObiE.E. and RamamurthiS., ‘The Firle eye magnet during the Industrial Revolution’, Journal of Trauma Injury, Infection, and Critical Care, 70 (2011), 519–52010.1097/TA.0b013e31820937fb21307756

96. Biddle, ‘The Health of Workers’, *op. cit.* (note 15), 97.

97. Mathias suggests that this aided comparative testing and experimentation in the armed forces during the late eighteenth century, *op. cit.* (note 49), 269.

98. Field, *op. cit.* (note 34), 15–16.

99. Mathias, *op. cit.* (note 49).

100. ChartersErica, ‘“The intention is certain noble”: The Western Squadron, medical trials, and the Sick and Hurt Board during the Seven Years War (1756–63)’, in D.B. Haycock and S. Archer (eds), Health and Medicine at Sea 1700–1900 (Woodbridge: Boydell Press, 2009), 19–37

101. By the nineteenth century, prevention was a well-established tenet of naval medicine: Patricia. K. Crimmin, ‘British naval health, 1700–1800: Improvement over time?’, in G.L. Hudson (ed.), *British Military and Naval Medicine 1600–1830* (Amsterdam: Rodopi, 2007), 183–200.

102. RNM 1983/621–2, 13/1–2, Office Files 1865–79, Admiralty circular 20 April 1865.

103. Examples can be found in NMM POR/J/6, memo 8 December 1828; letter August 1849.

104. NMM POR/J/6, letter 10 March 1849.

105. Lloyd and Coulter, *op. cit.* (note 9), chs VI, XII and XIII. Naval surgeons also wrote extensively on the subject: Bartrip, *op. cit.* (note 55), 14. Mathias argues that the work and findings of military and naval doctors during the eighteenth century were an important precursor to the nineteenth-century public health movements: Mathias, *op. cit.* (note 49).

106. RodgerN.A.M., The Command of the Ocean (London: Penguin, 2005), 486

107. A good introduction to this literature can be found in Bernard Harris, *The Origins of the British Welfare State: Social Welfare in England and Wales, 1800–1945* (Basingstoke: Palgrave Macmillan, 2004).

